# Growth-Promoting Effects of Dark Septate Endophytes Fungus *Acrocalymma* on Tomato (*Solanum lycopersicum*)

**DOI:** 10.3390/jof11070510

**Published:** 2025-07-07

**Authors:** Xiaoxiao Feng, Ying Jin, Zhupeiqi Zhong, Yongli Zheng, Huiming Wu

**Affiliations:** 1 The Rural Development Academy, Zhejiang University, Hangzhou 310058, China; xxvon@zju.edu.cn; 2 State Key Laboratory of Rice Biology and Breeding, Institute of Pesticide and Environmental Toxicology, College of Agriculture and Biotechnology, Zhejiang University, Hangzhou 310058, China; 3 College of Advanced Agriculture Sciences, Zhejiang Agricultural and Forestry University, Hangzhou 311300, China; jinying1312@outlook.com (Y.J.); 15869247671@163.com (Z.Z.); 4 Zhejiang Provincial Department of Agriculture and Rural Affairs, Hangzhou 310003, China

**Keywords:** dark septate endophytes (DSEs), *Acrocalymma*, plant growth promotion

## Abstract

This study investigates the potential role of *Acrocalymma* dark septate endophytic (DSE) fungi in promoting the growth of *Solanum lycopersicum* (tomato). Recognized as important symbionts that enhance plant growth and resilience under stress, particularly *Acrocalymma* species, DSE fungi were the focus of this investigation. Specifically, four stains isolated from gramineous plant roots (*Acrocalymma* sp. E00677, *Acrocalymma vagum* E00690, *Acrocalymma chuxiongense* E01299A, and *Acrocalymma chuxiongense* E01299B) were examined. Morphological characteristics were observed using three different media, confirming typical DSE traits such as dark pigmentation and septate hyphae. Phylogenetic analysis using six genetic markers (ITS, LSU, SSU, *tef*1, *rpb*2, and *tub*2) placed the strains within the *Acrocalymma* genus. Co-culture test and physiological index measurements showed that all strains significantly enhanced root development, as evidenced by an increased root-to-shoot ratio and a higher number of lateral roots. Additionally, the *Acrocalymma* DSE strains elevated chlorophyll a, chlorophyll b, and total chlorophyll content, suggesting improved photosynthetic efficiency. Anthocyanin levels were also increased in the tomato leaves, indicating enhanced antioxidative defense mechanisms. Among these strains, *Acrocalymma vagum* E00690 exhibited the most substantial effect on root activity. The widespread presence of 325 *Acrocalymma* isolates from 25 countries underscores its broad ecological adaptability. These findings suggest that *Acrocalymma* DSE fungi positively influence tomato growth, with potential implications for improving plant resilience under environmental stress. This study highlights the importance of further exploring DSEs, particularly *Acrocalymma* fungi, to better understand their ecological roles in agricultural practices, particularly in tomato cultivation.

## 1. Introduction

Tomato (*Solanum lycopersicum* L.) is one of the most widely cultivated and economically important crops worldwide, with a global production exceeding 180 million metric tons in 2022 (http://www.fao.org/faostat, accessed on 28 May 2025). As a key horticultural crop, it plays a vital role in global food security and agricultural economies, supporting both fresh markets and the processed food industry [1,2]. Due to its high economic value and widespread cultivation, tomato has become a major focus of agricultural research, particularly in efforts to enhance yield, improve stress tolerance, and promote sustainable production [3,4,5,6]. However, tomato cultivation is frequently challenged by various biotic and abiotic stresses [7,8,9,10]. Pathogens such as *Fusarium oxysporum* and *Ralstonia solanacearum* pose serious threats to plant health, leading to significant yield losses [11,12,13,14,15,16]. Additionally, environmental factors like drought and soil salinity further constrain tomato production, underscoring the urgent need for sustainable strategies to enhance plant growth and resilience [17,18,19]. In this regard, beneficial microorganisms, particularly dark septate endophytes (DSEs), have gained increasing attention for their potential to improve plant health, enhance stress tolerance, and promote overall agricultural sustainability [20,21,22,23].

Dark septate endophytes (DSEs), a major group of *Ascomycetous* endophytes, are characterized by melanized septate hyphae and microsclerotia in plant roots [18,24,25,26]. These root-associated fungi are widely distributed across various crops, where they contribute to plant growth by improving nutrient uptake, modulating phytohormones, and increasing stress tolerance [3,17,27,28,29]. Notably, DSEs have been reported in wheat, rice, maize, and tomato, where they enhance biomass accumulation and disease resistance [30,31,32,33,34,35,36,37,38]. Although less studied than mycorrhizal fungi, growing evidence suggests that DSE play a significant role in sustainable agriculture. Among them, the genus *Acrocalymma* has been identified as a plant endophyte with potential growth-promoting effects [26,39,40,41,42]. However, its specific function in crop production remains largely unexplored, warranting further investigation.

The beneficial effects of DSEs on plant growth can be attributed to multiple mechanisms. First, DSEs enhance nutrient acquisition by solubilizing insoluble forms of essential elements such as nitrogen and phosphorus, thereby improving plant nutrient availability [43,44,45,46]. Second, they influence phytohormone levels, including auxins, gibberellins, and abscisic acid, which regulate root architecture and overall biomass accumulation [47,48,49,50]. Third, DSEs contribute to abiotic stress tolerance by enhancing water use efficiency and modulating antioxidant enzyme activities, thereby improving plant resilience to drought and salinity [51,52,53]. Additionally, some DSEs can suppress pathogens through competition or by inducing systemic resistance in host plants [36,54,55]. Collectively, these mechanisms make DSEs promising candidates for sustainable agricultural practices aimed at improving crop productivity under both optimal and stressful conditions.

## 2. Materials and Methods

### 2.1. Sample Collection and Fungal Isolation

Plant samples (*Eleusine indica* and *Oryza meyeriana*) were collected at 101.576° E, 21.555° N in the core area of the Naban River Nature Reserve, Xishuangbanna, Yunnan Province, China. The roots of gramineous plants were surface sterilized in 75% ethanol for 30 s, followed by 1% sodium hypochlorite for 3 min, and then rinsed three times with sterilized water to remove any residual disinfectants. The sterilized root segments were cut into approximately 0.5 cm pieces and placed on potato dextrose agar (PDA) supplemented with chloramphenicol (50 mg/L) to inhibit bacterial contamination [56]. The plates were incubated at 25 °C in the dark for 5–7 days, and emerging fungal colonies were transferred onto fresh PDA plates for purification [57]. Morphologically distinct fungal isolates were further subcultured until pure cultures were obtained.

### 2.2. Morphological Characterization

The purified fungal isolates were cultured on potato dextrose agar (PDA; 10 g potato infusion powder, 20 g dextrose, 0.1 g chloramphenicol, and 13 g agar per liter), malt extract agar (MEA; 34 g malt extract and 15 g agar per liter), and oatmeal agar (OMA; 30 g oatmeal and 15 g agar per liter) and incubated at 25 °C for 7–14 days to observe colony characteristics, including color, texture, and growth pattern. Colony appearances were photographed using a Canon EOS 700D digital camera (Canon Inc., Tokyo, Japan). Microscopic (Leica DM2500, Leica Microsystems, Wetzlar, Germany) features were examined by observation under a light microscope (×400, ×1000 magnification). Morphological features such as hyphal structure and conidia were recorded [57].

### 2.3. Molecular Identification

The isolated DSE was identified as *Acrocalymma* spp. through molecular characterization. Fresh fungal mycelia from pure cultures grown on PDA at 25 °C for 5 to 7 days were used for genomic DNA extraction, following the protocol provided by the DNA extraction kit (Zhejiang Shangya Biotechnology Co., Ltd., Hangzhou, China). PCR amplification was conducted as described by Liu et al. [58], targeting multiple genetic regions, including the internal transcribed spacer (ITS) [59], partial large subunit nuclear rDNA (LSU) [60], partial small subunit nuclear rDNA (SSU) [59], RNA polymerase II second largest subunit (*rpb*2) [61], translation elongation factor 1-alpha (*tef*1) [61,62], and β-tubulin (*tub*2) [61,63], using the primers listed in Table 1. PCR reactions were performed in a Veriti 96-well thermal cycler (Thermo Fisher Scientific Inc., Shanghai, China) with a total volume of 20 μL, containing 2× Phanta Master Mix (Vazyme, Nanjing, China), 0.1 μM of each primer, and 10 ng of genomic DNA. The amplified products were purified and sequenced by Zhejiang Shangya Biotechnology Co., Ltd. (Hangzhou, China). PCR products were purified and sequenced, and the obtained sequences were compared with reference sequences in the NCBI GenBank database using BLAST (version 2.16.0) (https://blast.ncbi.nlm.nih.gov/Blast.cgi, accessed on 15 May 2025) analysis for species confirmation. The identified fungal strains were deposited in the General Microbiology Center of the Guangdong Microbial Culture Collection Center (GDMCC), Guangzhou, China, under the preservation numbers CDMCC No. 63140 (*Acrocalymma* sp. E00677), CDMCC No. 63141 (*Acrocalymma vagum* E00690), CDMCC No. 63143 (*Acrocalymma chuxiongense* E01299A), and CDMCC No. 63153 (*Acrocalymma chuxiongense* E01299B).

### 2.4. Phylogenetic Analyses

Phylogenetic analysis was performed using the PhyloSuite platform v1.2.3 [64]. In brief, sequences from the ITS, LSU, SSU, *rpb*2, *tef*1, and *tub*2 regions were aligned using MAFFT v7.505 [65], followed by trimming and concatenation. Taxa lacking one or more loci were retained in the concatenated alignment. Missing sequences were treated as gaps (‘?’), and phylogenetic inference was conducted using partition-aware models in IQ-TREE v2.2.0 and MrBayes v3.2.7a, which can accommodate incomplete data matrices without excluding taxa. The optimal partition model was determined using ModelFinder v2.2.0 [66] based on the Bayesian Information Criterion (BIC) and Akaike Information Criterion (AIC). Maximum likelihood (ML) phylogenetic trees were constructed with IQ-TREE [67] under the edge-linked partition model, with 5000 ultrafast bootstrap replicates [68]. Bayesian inference (BI) analysis was conducted in MrBayes [69] using the same partition model, with the first 25% of sampled data discarded as burn in. The generated phylogenetic trees were visualized in FigTree v1.4.3, with ML bootstrap values (MLBS) above 75% and Bayesian posterior probabilities (BYPP) exceeding 0.95 displayed at the nodes. The final phylogram was edited using Adobe Illustrator v.27.5 (Adobe Systems Inc., San Jose, CA, USA).

### 2.5. Evaluation of Plant Growth Promotion by Endophytic Strains Under Greenhouse Conditions

To evaluate the growth-promoting effects of *Acrocalymma* spp., a co-culture experiment was conducted using tomato (*Solanum lycopersicum* L.) ‘Daianna’ seedlings. Tomato seeds were surface sterilized with 75% ethanol for 30 s, followed by 0.5% sodium hypochlorite for 15 min, and then rinsed three times with sterile distilled water [10]. After sterilization, seeds were placed on sterilized and dried filter paper and allowed to air dry under a laminar flow hood. Sterilized seeds were germinated on moist filter paper in Petri dishes at 25 °C in the dark for 3 days.

For fungal inoculation, *Acrocalymma* spp. was first cultured on potato dextrose agar (PDA) at 25 °C for 7 days. Mycelial plugs (5 mm diameter) were then placed near the roots of 7-day-old tomato seedlings grown in sterilized vermiculite under controlled conditions. Non-inoculated seedlings served as controls. Plants were placed in a 1/2 MS medium (Murashige & Skoog) plate. All the inoculation processes were carried out on a clean bench, and all the dishes were kept in a growth chamber with a 16 h/8 h photoperiod, temperature of 25 °C/22 °C (day/night), and 60% mean air relative humidity. The duration of the stress experiment was 3 weeks.

### 2.6. Physiological Indexes Measurement

To evaluate the effects of *Acrocalymma* isolates on tomato growth, physiological and morphological parameters were measured after 21 days of co-cultivation. Tomato seedlings were carefully uprooted, and lateral roots were gently washed with sterile distilled water to remove residual substrate.

#### 2.6.1. Morphological Measurements

Growth parameters, including shoot height, root length, root number, shoot biomass, root biomass, and root-to-shoot ratio, were recorded. Root-to-shoot ratio was calculated as root fresh weight divided by shoot fresh weight.

#### 2.6.2. Physiological and Biochemical Analyses

Chlorophyll and carotenoid content were determined using the 95% ethanol extraction method, with absorbance measured at 470 nm, 649 nm, and 665 nm [70]. Anthocyanin content was quantified following the acidic methanol extraction method, with absorbance recorded at 530 nm and 637 nm [71]. Root vitality was assessed using the TTC (triphenyl tetrazolium chloride) reduction assay, with absorbance measured at 485 nm [72]. Each treatment was replicated three times, and statistical analyses were performed to evaluate the effects of fungal inoculation on tomato growth and physiology.

### 2.7. Geographic Coordinates Analysis

To comprehensively analyze the diversity and geographical distribution of *Acrocalymma* species, a meta-analysis was conducted using available records from public databases. Geographic coordinate information of representative *Acrocalymma* species and closely related genera were retrieved from the NCBI GenBank database. Geographic coordinates, when available, were mapped to visualize the worldwide occurrence of *Acrocalymma* species [73]. The distribution patterns were further processed and displayed using the online platform ChiPlot (https://www.chiplot.online/, accessed on 6 April 2025) to generate heatmaps and spatial distribution graphs. This meta-analysis provides a broader perspective on the ecological and evolutionary aspects of *Acrocalymma*.

### 2.8. Statistics and Analysis

The correlations among the different variables were analyzed using the Pearson coefficient. Statistical data were plotted using GraphPad Prism 9. All experimental data, including root biomass, shoot biomass, root-to-shoot ratio, lateral roots number, chlorophyll a, chlorophyll b, total chlorophyll, anthocyanin content, and root vitality, were analyzed using a two-way analysis of variance (ANOVA) to evaluate the effects of *Acrocalymma* inoculation on tomato growth. Treatment (inoculated vs. non-inoculated) and individual fungal isolates were considered as independent factors. Post hoc multiple comparisons were performed using Tukey’s honestly significant difference (HSD) test to determine significant differences among treatments. All statistical analyses were conducted using SPSS (v.26.0, IBM, New York, NY, USA), with significance set at *p* < 0.05. Results are presented as mean ± standard error (SE), and graphs were generated using GraphPad Prism (v.9.0, GraphPad Software, Boston, MA, USA).

## 3. Results

### 3.1. Morphological Characteristics of Isolated DSE

**Figure 1:**
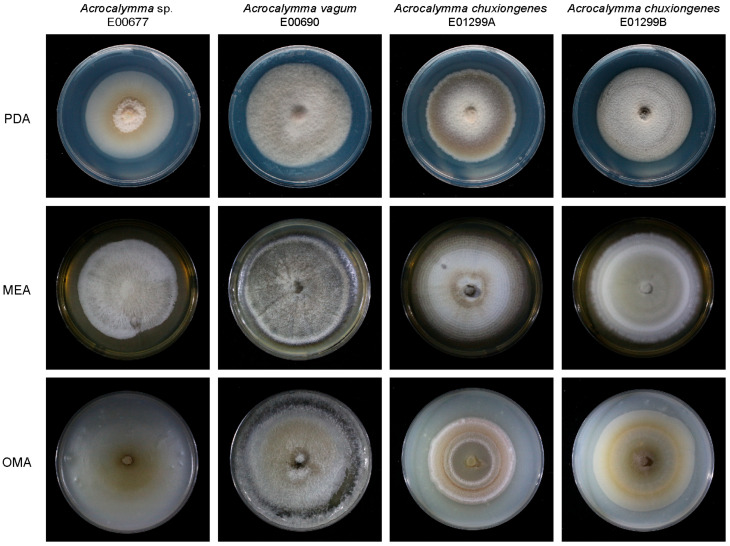
*Acrocalymma* DSE (*Acrocalymma* sp. E00677, *Acrocalymma vagum* E00690, *Acrocalymma chuxiongense* E01299A, and *Acrocalymma chuxiongense* E01299B) colony morphology on PDA, MEA, and OMA.

In total, 16 fungal isolates were obtained from the roots of gramineous plants collected in the Naban River Nature Reserve, Xishuangbanna, Yunnan Province, China. Combining morphological characteristics with ITS sequences, the 16 fungal isolates were identified as 4 fungal strains, which belong to *Acrocalymma*. The colonies grown on PDA, MEA, and OMA exhibited slow to moderate growth, with darkly pigmented, cottony to velvety textures (Figure 1). On the PDA plates, isolate E00677 exhibited a compact growth pattern, closely adhering to the medium surface, with fine hyphae forming a tough, resilient colony surface. In contrast, isolates E00690, E01299A, and E01299B displayed dense hyphal growth with a grayish, short-velvety appearance. On MEA, E00677 formed white, long-filamentous hyphae, while E00690 developed grayish-brown hyphae with a white colony margin. Isolate E01299A produced white to light brown colonies with distinct concentric ring patterns, whereas E01299B exhibited a gray, felt-like colony morphology. On OMA, E00677 displayed sparse, light brown hyphae, whereas E00690 formed a grayish-white, short-velvety colony. E01299A and E01299B exhibited light brown to brown colonies with concentric ring patterns. The colonies grew slowly, only reaching 6 cm in diameter after 1 month of culturing under 25 ± 2 °C in 16/8 light and darkness. No sexual or asexual spores were observed.

### 3.2. Phylogenetic Analysis

**Figure 2:**
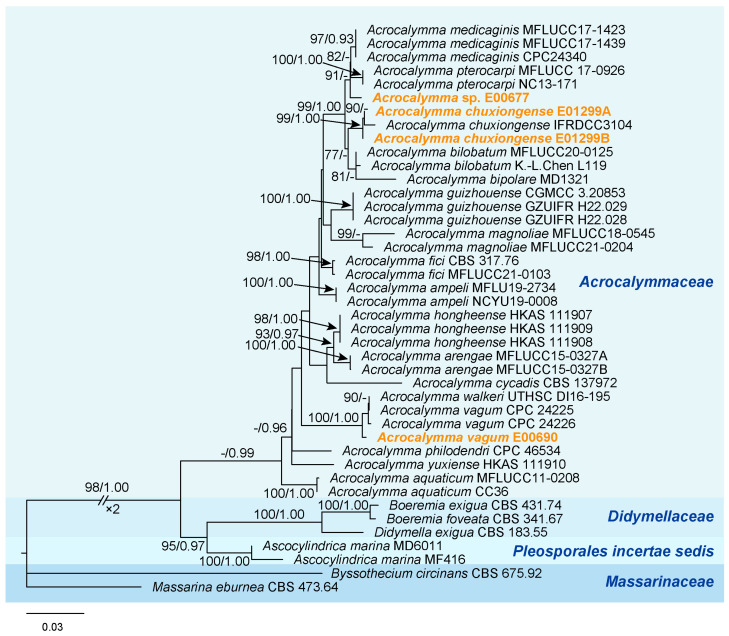
Phylogenetic tree generated by a maximum likelihood analysis based on a concatenated dataset of ITS, LSU, SSU, *rpb*2, *tef*1, and *tub*2. Maximum likelihood bootstrap values ≥ 75% (left) and Bayesian inference posterior probability ≥ 0.95 (right) are indicated at nodes (MLBS/BYPP). *Byssothecium circinans* CBS 675.92 and *Massarina eburnea* CBS 473.64 were chosen as the outgroup.

The concatenated alignment used for phylogenetic analysis included 37 sequences (Table 2), consisting of 6633 positions with 1323 distinct patterns, 938 parsimony-informative sites, 552 singleton sites, and 5143 constant sites, including gaps. The aligned regions were distributed as follows: SSU (positions 1–1423), LSU (1424–2794), ITS (2795–3281), *tef*1 (3282–4223), *rpb*2 (4224–5289), and *tub*2 (5290–6633). Model selection using ModelFinder identified GTR+I+G and GTR+F+I+G4 as the best-fit models based on the Bayesian Information Criterion (BIC) and Akaike Information Criterion (AIC). Since the Maximum Likelihood (ML) and Bayesian Inference (BI) analyses generated highly consistent topologies, only the ML tree is presented, with ML bootstrap support (MLBS) and Bayesian posterior probabilities (BYPP) displayed at the nodes. The phylogenetic analysis confirmed that all four fungal isolates in this study belong to the genus *Acrocalymma* (Figure 2). Specifically, *Acrocalymma* sp. E00677 formed an independent branch closely related to *A. medicaginis* and *A. pterocarpi*, with strong bootstrap support (100% MLBS, 1.00 BYPP), suggesting that E00677 may represent a novel species within the genus *Acrocalymma*. *A. chuxiongense* E01299A and *A. chuxiongense* E01299B clustered together in a distinct lineage with *A chuxiongense*. *A. vagum* E00690 formed an independent branch associated with *A. vagum* and *A. walkeri*. These three branches together constituted an independent, well-supported clade (100% MLBS, 1.00 BYPP), indicating robust phylogenetic placement. *Byssothecium circinans* CBS 675.92 and *Massarina eburnea* CBS 473.64 were chosen as the outgroup.

### 3.3. Effect of Acrocalymma Inoculation on Physiological Indexes

**Figure 3:**
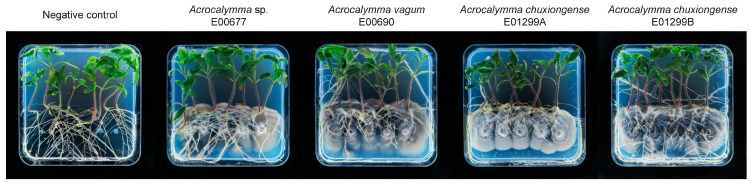
Tomato seedlings co-cultivated with *Acrocalymma* DSE under controlled conditions.

The co-cultivation experiments demonstrated that four *Acrocalymma* isolates significantly promoted the growth and physiological performance of *Solanum lycopersicum* ‘Daianna’ seedlings, with distinct effects observed among different isolates (Figure 3).

#### 3.3.1. Root Development and Biomass Allocation

**Figure 4:**
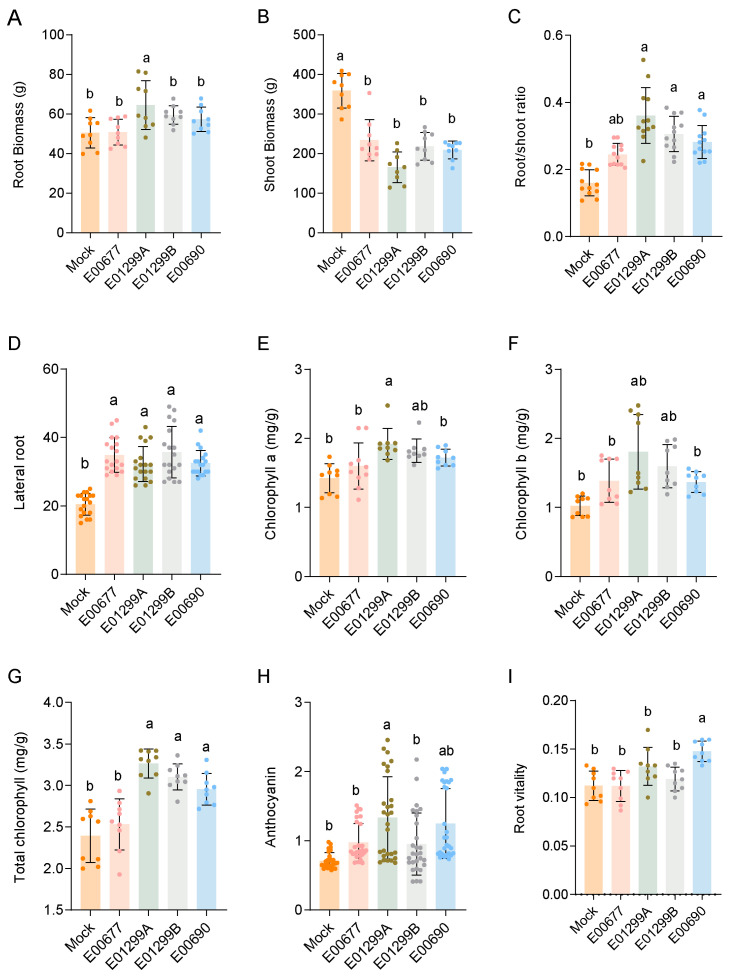
Effects of *Acrocalymma* endophyte inoculation on physiological traits of *Solanum lycopersicum* ‘Daianna’ seedlings. (**A**) Root biomass; (**B**) shoot root biomass; (**C**) root/shoot ratio; (**D**) lateral root; (**E**) chlorophyll a content; (**F**) chlorophyll b content; (**G**) total chlorophyll content; (**H**) anthocyanin content; (**I**) root vitality assessed via TTC reduction assay. Tomato seedlings were inoculated with four *Acrocalymma* isolates (*Acrocalymma* sp. E00677, *Acrocalymma vagum* E00690, *Acrocalymma chuxiongense* E01299A, and *Acrocalymma chuxiongense* E01299B) and compared to a non-inoculated control. Data represent the mean ± standard error (*n* = X, specify number of replicates). Different lowercase letters above the bars indicate statistically significant differences among treatments at *p* < 0.05, based on one-way ANOVA followed by Tukey’s HSD test. These results demonstrate that specific *Acrocalymma* strains significantly promote tomato seedling growth by enhancing root architecture, chlorophyll accumulation, anthocyanin levels, and root vitality.

*Acrocalymma* sp. E00677 significantly enhanced lateral root formation, with the number of lateral roots increasing by 53.77% compared with the non-inoculated control (1.54-fold of the control) (Figure 4D). This indicates an expanded root system that improves nutrient and water absorption efficiency. Although *Acrocalymma* sp. E00677 slightly reduced the average root biomass (by 1.34%), it significantly increased the root-to-shoot ratio by 53.90% (1.54-fold of the control), suggesting enhanced root development relative to shoot growth, which contributes to better plant stability and resource uptake (Figure 4A,B). *A. chuxiongense* E01299A and *A. chuxiongense* E01299B also promoted root system development, with *A. chuxiongense* E01299A increasing lateral root number by 36.66% (1.37-fold of the control) and *A. chuxiongense* E01299B by 53.77% (1.54-fold of the control). Notably, *A. chuxiongense* E01299A demonstrated the strongest effect on root-to-shoot ratio, increasing it by 125.04% (2.25-fold of the control), while *A. chuxiongense* E01299B increased it by 90.17% (1.90-fold of the control), indicating a shift in biomass allocation favoring root development (Figure 4C).

#### 3.3.2. Photosynthetic Pigments and Stress-Related Compounds

Inoculation with *Acrocalymma* sp. E00677 significantly increased chlorophyll content, with chlorophyll a, chlorophyll b, and total chlorophyll levels rising by 9.28%, 18.18%, and 7.76%, respectively, compared with the control (Figure 4E–G). *A. chuxiongense* E01299A induced even greater increases, elevating chlorophyll a by 32.12%, chlorophyll b by 50.85% (1.51-fold of the control), and total chlorophyll by 34.39%. *A. chuxiongense* E01299B also enhanced chlorophyll accumulation, increasing chlorophyll a by 23.64%, chlorophyll b by 31.50% (1.31-fold of the control), and total chlorophyll by 23.51%, indicating improved photosynthetic efficiency.

#### 3.3.3. Anthocyanin Accumulation

Anthocyanin content, an indicator of oxidative stress tolerance, significantly increased following inoculation. *Acrocalymma* sp. E00677 treatment led to a 37.52% increase, whereas *A. chuxiongense* E01299A resulted in the highest accumulation (87.18%, 1.87-fold of the control), suggesting enhanced antioxidant potential. *A. chuxiongense* E01299B also increased anthocyanin levels by 33.75% (Figure 4H).

#### 3.3.4. Root Vitality

Root vitality assessed via the TTC reduction assay showed a moderate yet significant enhancement in *Acrocalymma chuxiongense* E01299A and *A. chuxiongense* E01299B treatments (5.77% increase), indicating improved root metabolic activity.

These findings confirm that *Acrocalymma* species, particularly *Acrocalymma* sp. E00677, *A. vagum* E00690, *A. chuxiongense* E01299A, and *A. chuxiongense* E01299B, act as growth-promoting endophytes in tomato. The observed improvements in root system architecture, chlorophyll content, anthocyanin accumulation, and root vitality highlight their potential for sustainable agriculture, offering promising applications for enhancing crop resilience and productivity under both optimal and stress conditions (Figure 4I).

### 3.4. Global Distribution and Ecological Adaptability on Acrocalymma

**Figure 5:**
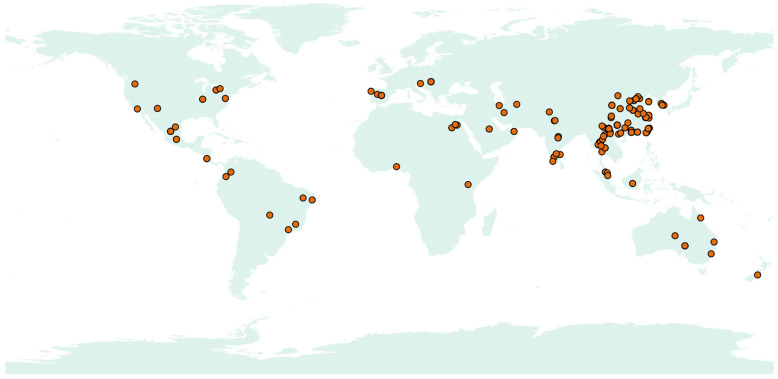
Global distribution of *Acrocalymma* dark septate endophytes (DSEs) based on publicly available sequence records.

A meta-analysis of publicly available sequence records revealed that *Acrocalymma* species are widely distributed across the globe. Our dataset included 325 isolates from 25 countries, underscoring the broad geographic range and ecological plasticity of this fungal genus (Table S1). Most reported isolations were derived from plant-associated environments, particularly the root tissues of various crops and woody plants. The global distribution map (Figure 5) shows that *Acrocalymma* occurs predominantly in Asia, Europe, and North America, with additional reports in Africa, South America, and Oceania, indicating that this genus thrives under diverse climatic and ecological conditions. Its presence across multiple biomes—from temperate forests to tropical agricultural systems—further supports its ecological flexibility and capacity to colonize a wide range of environmental niches. These findings highlight *Acrocalymma* as a globally distributed, endophytic, and plant-associated fungal genus with potential roles in plant health, stress resilience, and sustainable agriculture. Further research is warranted to explore its functional diversity and interactions with host plants across different ecosystems.

## 4. Discussion

Dark septate endophytes play a vital role in plant nutrient absorption, growth, and defense, forming an essential component of the ecosystem [74]. In this study, four DSE fungi were isolated from the roots of gramineous plants and identified as belonging to the genus *Acrocalymma.* Fungi of this genus have been previously reported to form symbiotic relationships with various host plants, acting as DSEs that contribute to enhanced plant growth and development, as well as increased tolerance to both biotic and abiotic stresses [22,26,32,39,40,41,42].

Phylogenetic analysis is currently one of the most important approaches for determining species classification. To further explore the phylogenetic relationships of the four *Acrocalymma* strains, we conducted a combined analysis of six genetic markers: ITS, LSU, SSU, *rpb*2, *tef*1, and *tub*2. The results confirmed that the four strains are closely related within the *Acrocalymma* genus, providing valuable insights into the evolutionary relationships within this group [75]. Notably, strain E00690 exhibited a close phylogenetic relationship with *Acrocalymma vagum*, suggesting a high degree of genetic similarity [76]. This close affinity may indicate shared ecological roles and functional properties, warranting further studies to explore their common traits and environmental adaptability [53]. Additionally, the phylogenetic analysis offers a solid foundation for future taxonomic studies within the *Acrocalymma* genus, contributing to a better understanding of its ecological significance and functional diversity [77,78,79].

An important approach to evaluating the functional roles of dark septate endophytic (DSE) fungi is by assessing their phenotypic effects on host plant growth. In addition, we assessed the growth-promoting effects of DSE strains E00677, E00690, E01299A, and E01299B by co-cultivating them with aseptically grown *Solanum lycopersicum* ‘Daianna’ seedlings. Our results demonstrated that all four DSE strains significantly enhanced tomato growth, particularly by promoting root development, as evidenced by an increased root-to-shoot ratio and a greater number of lateral roots. The increase in plant biomass suggests that DSE fungi may help host plants accumulate more organic matter, potentially enabling them to better withstand prolonged periods of high temperatures and drought stress—conditions frequently encountered in their natural environments [80]. Improvements were also observed in several physiological parameters of the shoots, including increased levels of chlorophyll a, chlorophyll b, and total chlorophyll, which suggests a role for these DSE fungi in enhancing photosynthetic capacity [81]. A higher chlorophyll content is typically associated with an enhanced ability to capture light energy, thereby improving the efficiency of photosynthesis [82]. This increased photosynthetic activity can lead to the greater production of sugars and other essential metabolites, ultimately supporting better growth and development [83]. Consequently, the observed increase in chlorophyll content in plants treated with these DSE strains may contribute to enhanced biomass accumulation and overall plant vigor.

Furthermore, all four strains elevated the anthocyanin content in the leaves, which may indicate an improved antioxidative defense system or enhanced tolerance to environmental stress [84]. Anthocyanins are known to act as protective pigments involved in mitigating oxidative damage caused by abiotic stress factors such as UV radiation, high temperatures, and drought. By scavenging reactive oxygen species (ROS), anthocyanins help protect plant cells from oxidative damage to membranes, proteins, and nucleic acids [85,86]. Additionally, anthocyanins may regulate plant growth by modulating hormonal responses and improving nutrient uptake [87]. Thus, the increased anthocyanin levels observed in the leaves could enhance stress resilience and overall plant health, promoting better growth under challenging environmental conditions.

Among the strains, *Acrocalymma vagum* E00690 exhibited the most pronounced effect on enhancing root activity. Increased root activity plays a crucial role in improving the plant’s ability to absorb water and nutrients from the soil, particularly under stress conditions. A more active root system can improve the uptake of essential minerals and organic compounds, supporting better plant growth and development [88]. Furthermore, increased root vigor is associated with greater root biomass and a more extensive root network, which can help plants establish stronger anchorage in the soil and access a wider soil volume for nutrient acquisition [38]. This enhanced root activity, therefore, contributes to overall plant vigor, improved stress tolerance, and potentially higher biomass accumulation, supporting optimal plant growth [89].

The role of DSEs in promoting tomato growth has been a topic of significant interest [90]. Our review reveals that *Acrocalymma* species are distributed in 25 countries worldwide, particularly in regions with extensive tomato cultivation. Based on the results of this study, we conclude that DSE fungi play a positive role in promoting host plant growth. Furthermore, this study highlights the need for an increased focus on endophytic fungi, particularly DSEs, to better understand their ecological roles and adaptive functions in tomato growth.

## 5. Conclusions

This study highlights the significant role of *Acrocalymma* strains as dark septate endophytic (DSE) fungi in promoting tomato growth. An examination of the growth morphology on different media confirmed that all four strains exhibit typical DSE colony characteristics, such as dark pigmentation and septate hyphae, supporting their classification as DSE fungi. A phylogenetic analysis based on six genetic markers (ITS, LSU, SSU, *rpb*2, *tef*1, and *tub*2) confirmed their placement within the *Acrocalymma* genus, offering insights into their evolutionary relationships. All four isolates significantly enhanced growth parameters, including root development, chlorophyll content, and anthocyanin levels, underscoring their positive impact on plant physiological functions. These findings suggest that DSE fungi, especially *Acrocalymma* species, could provide a natural strategy to enhance plant resilience under stress conditions. The widespread distribution of *Acrocalymma* across 25 countries further highlights the genus’s broad ecological adaptability. This study also emphasizes the need for a further exploration of endophytic fungi, particularly DSEs, to deepen our understanding of their ecological roles and explore their potential applications in sustainable agriculture, particularly in tomato cultivation.

## Figures and Tables

**Table 1:** Gene regions and primer pairs used in this study.

Gene Regions	Primers	Sequences of Primers 5′-3′	References
ITS	ITS5	GGAAGTAAAAGTCGTAACAAGG	(White et al., 1990) [59]
	ITS4	TCCTCCGCTTATTGATATGC	
LSU	LR0R	ACCCGCTGAACTTAAGC	(Rehner & Samuels, 1995) [60]
	LR5	ATCCTGAGGGAAACTTC	
SSU	SSU-SR1R	TACCTGGTTGATTCTGC	(White et al., 1990) [59]
	SSU-SR7	GTTCAACTACGAGCTTTTTAA	
*rpb*2	RPB2-5F2	GGGGWGAYCAGAAGAAGGC	(O’Donnell et al., 2007) [61]
	RPB2-7cR	CCCATRGCTTGYTTRCCCAT	
*tef*1	EF1-728F	CATCGAGAAGTTCGAGAAGG	(Carbone & Kohn, 1999) [62]
	EF2	GGARGTACCAGTSATCATG	(O’Donnell et al., 2007) [61]
*tub*2	T1	AACATGCGTGAGATTGTAAGT	(O’Donnell et al., 2007) [61]
	CYLTUB1R	AGTTGTCGG GACGGAAGAG	(Crous et al., 2006) [63]

**Table 2:** Reference strains information and GenBank accession numbers used in the phylogenetic analyses. A dash (“_”) indicates missing or unavailable accession numbers in the GenBank database.

Taxon	Strain/Voucher Number	Genbank Accession Number					
		ITS	LSU	SSU	*rpb*2	*tef*1	*tub*2
*Acrocalymma ampeli*	MFLU19-2734	MW063150	MW063211	MW079341	_	_	_
*Acrocalymma ampeli*	NCYU19-0008	MW063151	MW063212	MW079342	_	_	_
*Acrocalymma aquaticum*	MFLUCC11-0208	NR_121544	NG_042698	JX276953	_	_	_
*Acrocalymma aquaticum*	CC36	MT875395	MT875393	_	_	MT897894	_
*Acrocalymma arengae*	MFLUCC15-0327A	ON650154	ON650673	ON650177	ON734014	_	ON745966
*Acrocalymma arengae*	MFLUCC15-0327B	ON650155	ON650674	ON650178	ON734015	_	
*Acrocalymma bilobatum*	K.-L.Chen L119	KX034339	_	_	_	_	_
*Acrocalymma bilobatum*	MFLUCC20-0125	MT875396	MT875394	_	_	MT897895	_
*Acrocalymma bipolare*	MD1321	_	NG_075326	_	_	_	_
*Acrocalymma chuxiongense*	IFRDCC3104	ON595715	ON596248	_	_	_	_
*Acrocalymma chuxiongense*	E01299A	PV716432	PV731392	PV739235	PV763383	PV763379	PV763388
*Acrocalymma chuxiongense*	E01299B	PV716431	PV731393	PV739234	PV763384	PV763380	PV763387
*Acrocalymma cycadis*	CBS 137972	NR_137884	NG_057046	_	_	_	_
*Acrocalymma fici*	CBS 317.76	NR_137953	NG_057056	_	_	KP170663	KP170687
*Acrocalymma fici*	MFLUCC21-0103	MT864351	MT860429	_	_	_	_
*Acrocalymma guizhouense*	CGMCC 3.20853	OM838410	OM838474	OM838471	_	_	_
*Acrocalymma guizhouense*	GZUIFR H22.028	OM838411	OM838475	OM838472	_	_	_
*Acrocalymma guizhouense*	GZUIFR H22.029	OM838412	OM838476	OM838473	_	_	_
*Acrocalymma hongheense*	HKAS 111907	MW424763	MW424777	MW424792	_	_	_
*Acrocalymma hongheense*	HKAS 111908	MW424762	MW424776	MW424791	_	_	_
*Acrocalymma hongheense*	HKAS 111909	MW424761	MW424775	MW424790	_	_	_
*Acrocalymma magnoliae*	MFLUCC18-0545	OL413439	OK655819	OL331094	_	_	_
*Acrocalymma magnoliae*	MFLUCC21-0204	OL413440	OK655820	OL331095	_	_	_
*Acrocalymma medicaginis*	CPC 24340	KP170620	KP170713	_	_	_	_
*Acrocalymma medicaginis*	MFLUCC17-1423	MT214338	MT214432	MT214387	_	_	_
*Acrocalymma medicaginis*	MFLUCC17-1439	MT214339	MT214433	MT214388	_	_	_
*Acrocalymma philodendri*	CPC 46534	PQ498969	PQ499018		PQ497740	PQ497755	_
*Acrocalymma pterocarpi*	MFLUCC17-0926	MK347732	NG_066306	MK347840	MK434897	MK360040	_
*Acrocalymma pterocarpi*	NC13-171	LC517880	LC517881	_	_	_	_
*Acrocalymma* sp.	E00677	PV716433	PV731390	PV739232	PV763382	PV763377	PV763386
*Acrocalymma vagum*	E00690	PV716434	PV731391	PV739236	PV763381	PV763378	PV763385
*Acrocalymma vagum*	CPC24225	KP170635	_	_	_	_	_
*Acrocalymma vagum*	CPC24226	KP170636	_	_	_	_	_
*Acrocalymma walkeri*	UTHSC Dl16-195	LT796832	LN907338	_	LT796992	LT797072	LT796912
*Acrocalymma yuxiense*	HKAS 111910	_	MW424778	MW424793	_	_	_
*Ascocylindrica marina*	MD6011	_	KT252905	KT252907	_	_	_
*Ascocylindrica marina*	MF416	_	MK007123	MK007124	_	_	_
*Boeremia exigua*	CBS 431.74	FJ427001	EU754183	EU754084	GU371780	GU349080	FJ427112
*Boeremia foveata*	CBS 341.67	GU237834	GU237947	GU238203	MN983393	_	GU237509
*Didymella exigua*	CBS 183.55	NR_135936	NG_069119	NG_061065	EU874850	GCA_010094145.1	GCA_010094145.1
*Byssothecium circinans*	CBS 675.92	OM337536	GU205217	_	GCA_010015675.1	GU349061	GCA_010015675.1
*Massarina eburnea*	CBS 473.64	OM337528	GU301840	_	GU371732	GU349040	GCA_010093635.1

## Data Availability

The original contributions presented in this study are included in the article/Supplementary Materials. Further inquiries can be directed to the corresponding author.

## Supplementary Materials

The following supporting information can be downloaded at: https://www.mdpi.com/article/10.3390/jof11070510/s1, Table S1: map coordinates of *Acrocalymma*.
